# Keap1 Cysteine 288 as a Potential Target for Diallyl Trisulfide-Induced Nrf2 Activation

**DOI:** 10.1371/journal.pone.0085984

**Published:** 2014-01-28

**Authors:** Sanghyun Kim, Hee-Geum Lee, Sin-Aye Park, Joydeb Kumar Kundu, Young-Sam Keum, Young-Nam Cha, Hye-Kyung Na, Young-Joon Surh

**Affiliations:** 1 Tumor Microenvironment Global Core Research Center, College of Pharmacy, Seoul National University, Seoul, South Korea; 2 College of Pharmacy, Keimyung University, Daegu, South Korea; 3 College of Pharmacy, Dongguk University, Ilsan, South Korea; 4 College of Medicine, Inha University, Incheon, South Korea; 5 Department of Food and Nutrition, Sungshin Women's University, Seoul, South Korea; 6 Department of Molecular Medicine and Biopharmaceutical Sciences, Graduate School of Convergence Science and Technology, Seoul, South Korea; 7 Cancer Research Institute, Seoul National University, Seoul, South Korea; North Carolina State University, United States of America

## Abstract

Diallyl sulfide, diallyl disulfide, and daillyl trisulfide (DATS) are major volatile components of garlic oil. In this study, we assessed their relative potency in inducing antioxidant enzyme expression. Among the three organosulfur compounds, DATS was found to be most potent in inducing heme oxygenase-1 (HO-1) and NAD(P)H:quinone oxidoreductase-1 (NQO1) in human gastric epithelial (AGS) cells. Furthermore, DATS administration by gavage increased the expression of HO-1 and NQO1 in C57BL/6 mouse stomach. Treatment with DATS increased the accumulation of nuclear factor-erythroid-2-related factor-2 (Nrf2) in the nucleus of cultured AGS cells and in mouse stomach *in vivo*. The DATS-induced expression of HO-1 and NQO1 was abrogated in the cells transiently transfected with Nrf2-siRNA or in the embryonic fibroblasts from *Nrf2*-null mice, indicating that Nrf2 is a key mediator of the cytoprotective effects of DATS. Pretreatment of AGS cells with *N*-acetylcysteine or dithiothreitol attenuated DATS-induced nuclear localization of Nrf2 and the expression of HO-1 and NQO1. Cysteine-151, -273 and -288 of Kelch-like ECH-associated protein-1 (Keap1), a cytosolic repressor of Nrf2, have been considered to act as a redox sensor and play a role in Nrf2 activation. To determine whether DATS could inactivate Keap1 through thiol modification, we established cell lines constitutively expressing wild type-*Keap1* or three different mutant constructs in which cysteine-151, -273, or -288 of Keap1 was replaced with serine by retroviral gene transfer. DATS failed to activate Nrf2, and to induce expression of HO-1 and NQO1 only in *Keap1*-C288S mutant cells. LC-ESI-MS/MS analysis of recombinant Keap1 treated with DATS revealed that the peptide fragment containing Cys288 gained a molecular mass of 72.1 Da equivalent to the molecular weight of mono-allyl mono-sulfide. Taken together, these findings suggest that DATS may directly interact with the Cys288 residue of Keap1, which partly accounts for its ability to induce Nrf2 activation and upregulate defensive gene expression.

## Introduction

Cells display a hormetic response to reactive oxygen species (ROS) and other toxicants by inducing the expression of a battery of antioxidant and other cytoprotective proteins [1]. Heme oxygenase-1 (HO-1) and NAD(P)H:oxidoreductase-1 (NQO1) are two representative enzymes, that play key roles in protecting cells and tissues against oxidative stress, inflammation and tumor promotion [1], [2], [3]. These enzymes serve as a safeguard for gastric mucosa by protecting the cells from oxidative and proinflammatory insults. HO-1 catalyzes the breakdown of the porphyrin ring to produce free heme, biliverdin and carbon monoxide. Biliverdin is further converted to bilirubin by biliverdin reductase. Both bilirubin and carbon monoxide have been reported to protect against inflammatory and oxidative tissue damages [4], [5]. NQO1 not only catalyzes the reduction processes of various quinone electrophiles, but also exerts antioxidant activity by facilitating the inactivation of superoxides [6], [7], [8]. More recently, the essential role of NQO1 in maintaining homeostasis of gastric tissue has been suggested. Thus, the polymorphism of NQO1 was found to be associated with increased susceptibility to gastric carcinogenesis among the people in East Asia [9], [10].

The cytoprotective enzymes including HO-1 and NQO-1 commonly share a conserved *cis*-acting enhancer sequence called antioxidant response element (ARE), which is a primary binding site of nuclear factor-erythroid-2-related factor 2 (Nrf2), a stress-responsive transcription factor. Nrf2 is normally sequestered and gradually degraded by the Kelch like ECH-associated protein 1 (Keap1) and Cul3 E3 ubiquitin ligase complex in the cytoplasm through the proteasome-dependent pathway. In response to electrophilic/oxidative stress, however, Nrf2 bypasses this proteasomal degradation and enters the nucleus, where it binds to the ARE and transactivates the expression of genes encoding antioxidant enzymes [11], [12], [13], [14]. Several studies have suggested that mitogen-activated protein kinases (MAPKs) and Akt are involved in Nrf2 activation [15], [16], [17], [18], while others have demonstrated that the evasion of Nrf2 from proteasomal degradation and subsequent induction of Nrf2-mediated signaling are most likely to be regulated by the alterations in the structure of Keap1. The site-directed mutagenesis of certain cysteine residues of Keap1 suggests that among the cysteine residues, Cys151, Cys273 and Cys288 are essential for regulating Nrf2 activation. These conserved cysteine residues in Keap1 function as a cellular redox sensor and can be subjected to oxidative or covalent modification. Thus, various ROS or electrophiles which cause oxidation or covalent modification of the Keap1 cysteine residues can activate Nrf2 and thereby upregulate defensive gene expression [14], [19], [20], [21].

Numerous edible phytochemicals have been shown to activate Nrf2. The use of garlic as a remedy has long been practiced in many societies. Major bioactive principles of garlic oil include the organosulfur compounds (OSC), such as diallyl sulfide (DAS), diallyl disulfide (DADS) and diallyl trisulfide (DATS) [22]. Despite extensive research on the anti-inflammatory, cardioprotective and chemopreventive effects of garlic-derived OSCs, the underlying molecular mechanisms have not been clearly defined. Since the oxidative stress plays a key role in the pathogenesis of various chronic diseases, including cancer, the chemoprotective and chemopreventive effects of OSCs are thought to be associated with their antioxidant properties [23], [24], [25]. This prompted us to compare the relative ability of DAS, DADS and DATS to induce Nrf2-driven antioxidant gene expression in human gastric epithelial (AGS) cells.

## Materials and Methods

### Materials

DATS (98% pure) was purchased from LKT laboratories (Minneapolis, MN, USA). RPMI 1640 media and fetal bovine serum (FBS) were obtained from GIBCO BRL (Grand Island, NY, USA). Antibodies against Nrf2, Keap1, c-Jun-N-terminal kinase (JNK), extracellular signal-regulated kinase (ERK) and phospho-ERK were procured from Santa Cruz Biotechnology (Santa Cruz, CA, USA). The primary antibody against NQO1 was a product of Abcam (Cambridge, MA, USA). An antibody against HO-1 was obtained from Stressgen Biotechnologies Co. (Victoria, BC, Canada). Dichlorofluorescein diacetate (DCF-DA), mouse monoclonal Lamin B_1_ antibody, human specific Nrf2-siRNA (sense 5′-AAGAGUAUGAGCUGGAAAAACTT-3′; antisense 5′-GUUUUUCCAGCUCAUACUCUUTT-3′), TRIzol reagent and Stealth™ RNAi negative control duplexes were purchased from Invitrogen Life Technologies, Inc. (Carlsbad, CA, USA). Keap1-targeting siRNA (sense 5′- GGCCUUUGGCAUCAUGAACUU-3′; antisense 5′-GUUCAUGAUGCCAAAGGCCUU-3′) and the scrambled siRNA (sense 5′-GACGAGCGGCACGUGCACAUU-3′; antisense 5′-UGUCGACGUGCCGCUCGUCUU′3′) were provided by Genolution Pharmaceuticals, Inc. (Seoul, Korea). DAS, DADS, *N*-acetyl-L-cysteine (NAC), dithiothreitol (DTT) and an antibody against actin were purchased from Sigma-Aldrich Co. (St Louis, MO, USA). Anti-Akt, anti-phospho-Akt and phosphor-JNK antibodies were from Cell Signaling Technology (Beverly, MA, USA). LY294002 and SB203580 were purchased from Calbiochem (San Diego, CA, USA). Polyvinylidene difluoride membranes were supplied from Gelman Laboratory (Ann Arbor, MI, USA). The enhanced chemiluminescence (ECL) detection kit was obtained from Amersham Pharmacia Biotech (Buckinghamshire, UK). The bicinchoninic acid (BCA) protein assay reagent was a product of Pierce Biotechnology (Rockfold, IL, USA).

### Cell culture

AGS (ATCC CRL1739) cells were obtained from American Type Culture Collection. Cells were cultured in RPMI 1640 medium supplemented with 10% FBS, 100 units/ml penicillin G, 100 µg/ml streptomycin sulfate and 250 ng/ml amphotericin B (Sigma-Aldrich, St. Louis, MO, USA) under humidified atmosphere of 5% CO_2_.

### Animal studies

Fifteen 6-week old female C57BL/6 mice were purchased from Orient Bio (Sungnam-si, South Korea). Mice weighing 16 to 20 g were randomly grouped into three and caged by group (*n* = 5 per treatment group in three separate cages). Mice were housed under specific pathogen-free conditions in climate-controlled quarters (24°C at 50% humidity) with a 12-h light/12-h dark cycle. Animals were given standard animal diet and water *ad libitum*. The control mice were given 0.1 ml of 0.05% sodium carboxymethyl cellulose (CMC) as a vehicle by gavage. Mice in the other two groups were received DATS (0.5 or 2 mg in 0.1 ml of CMC per mouse) by gavage every other day for two weeks. Animals were checked daily for water consumption and body weight according to the institution protocol (SOP Education No. of Seoul National University: 2006-06-62). All the mice were euthanized by cervical dislocation after 2 weeks of vehicle or DATS treatment. All experimental protocols were approved by the Animal Care and Use Committee (ACUC) of Seoul National University, (permit number: SNU-200909-14).

### Preparation and maintenance of mouse embryonic fibroblasts (MEF)

The *Nrf2* wild type (*Nrf2* ^+/+^) and *Nrf2*-null (*Nrf2* ^−/−^) mice were originally provided by Dr. Jeffery Johnson, University of Wisconsin, Madison, USA. After in-house breeding, the *Nrf2* ^−/−^, *Nrf2* ^+/−^ and wild-type mice were maintained in the animal quarters and were housed in a 12-h light/dark cycle. They were fed standard rodent chow and given water *ad libitum*. Male and female *Nrf2^+^* ^/−^ mice were mated, and embryos were obtained at the day 13.5 after pairing under aseptic conditions. The heads of the embryos were used to confirm the *Nrf2* genotype by polymerase chain reaction, and the embryo bodies were minced into small pieces and cultured in high glucose DMEM supplemented with 10% FBS and kept at 37°C with 5% CO_2_.

### Western blot analysis

Cells, treated with or without OSCs for indicated time periods, were washed with phosphate-buffered saline (PBS) and incubated with cell lysis buffer [50 mM Tris–HCl (pH 7.4), 150 mM NaCl, 25 mM NaF, 20 mM ethylenediaminetetraacetic acid, 1 mM DTT, 1 mM Na_3_VO_4_, 0.5% Triton X-100 and protease inhibitor cocktail tablets] for 1 h on ice, followed by centrifugation at 13,000 rpm for 15 min. The protein concentration of the supernatant was measured by using the BCA reagents. The protein samples were solubilized with sodium dodecyl sulfate (SDS)–polyacrylamide gel electrophoresis sample loading buffer and boiled for 5 min. Proteins were electrophoresed on 7 or 10% SDS–polyacrylamide gel and transferred to polyvinylidene difluoride membranes. The blots were then blocked with 5% fat-free dry milk-TBST (TRIS-buffered saline containing 0.1% Tween-20) buffer for 1 h at room temperature and incubated with primary antibodies in 3% fat-free dry milk-TBST. Following three washes with TBST, the blots were incubated with horseradish peroxidase-conjugated secondary antibody in 3% fat-free dry milk-TBST for 1 h at room temperature. The blots were rinsed again three times with TBST, and the transferred proteins were incubated with ECL substrate solution (Amersham Pharmacia Biotech, Piscataway, NJ, USA) for 1 min according to the manufacturer's instruction and visualized with LAS 4000 (Fuji film, Japan).

### Preparation of cytosolic and nuclear extracts

Cells were pelleted by centrifugation after washing with cold PBS and suspended in ice-cold hypotonic buffer A [10 mM HEPES, pH 7.9, 1.5 mM MgCl_2_, 10 mM KCl, 0.5 mM DTT and 0.2 mM phenylmethylsulfonyl fluoride (PMSF)]. Following incubation in an ice bath for 15 min, cells were centrifuged again, and the supernatant was collected as a cytosolic fraction. The remaining cell pellets were resuspended in ice-cold buffer C containing 20 mM HEPES (pH 7.9), 20% glycerol, 420 mM NaCl, 1.5 mM MgCl_2_, 0.2 mM EDTA, 0.5 mM DTT, and 0.2 mM PMSF and were incubated at 0°C for 2 h. After vortex mixing, the resulting suspension was centrifuged (12,000 rpm, 15 min), and the supernatant was collected as a nuclear extract and stored at −70°C. The protein concentration was determined by the Bradford method using the Bio-Rad Protein Assay Kit (Bio-Rad Laboratories, Hercules, CA, USA).

### Immunocytochemistry of Nrf2

To demonstrate the nuclear translocation of Nrf2, immunocytochemistry was performed. AGS cells were plated on the chamber slide and treated with DATS or vehicle alone. After fixation with 10% neutral-buffered formalin solution for 30 min at room temperature, samples were incubated with blocking agents [0.1% Tween-20 in PBS containing 5% bovine serum albumin], washed with PBS and then incubated with a diluted (1∶100) primary antibody for overnight at 4°C. After washing with PBS, samples were incubated with a diluted (1∶1000) FITC-goat anti-rabbit IgG secondary antibody for 1 h and examined under a confocal microscope (Leika, Germany).

### Measurement of ROS

The intracellular level of ROS in AGS cells treated with DAS, DADS, or DATS was determined by using DCF-DA, a fluorescence-generating probe. Cells were incubated with with 20 µM of DAS, DADS, or DATS in the presence or absence of NAC (1 mM) for 3 h and rinsed with PBS. The treated cells were then incubated with 10 µM DCF-DA for 30 min at 37°C. The cells were washed once with Hanks' balanced salt solution (HBSS) and the ROS-mediated oxidation of DCF-DA to the fluorescent compound DCF was assessed by flow cytometric analyses. The cells were analyzed using a FACS Calibur flow cytometer (BD Biosciences, San Jose, CA) using the FL-1 channel (515–545 nm). The mean fluorescence intensity was analyzed using CellQuest Pro 4.0.2 software.

### Nrf2-siRNA transient transfection

AGS cells plated in 60-mm dishes were transfected with Nrf2-siRNA or Nrf2-negative control siRNA for 48 h. The transfected cells were treated with DATS for additional 6 h, followed by Western blot analysis. In another experiment, cells were transfected with scrambled or Keap1 siRNA for 24 h and treated with DATS for additional 2 h followed by preparation of whole cell lysate for immunoblot analysis.

### Reverse transcriptase-polymerase chain reaction (RT-PCR)

Total RNA was isolated from AGS cells using TRIzol®. Total RNA (1 µg) was used for the cDNA synthesis using random primers. RT-PCR was performed following standard procedures. PCR conditions for *Nrf2, HO-1* and *NQO1* as well as for the housekeeping gene, glyceraldehyde-3-phosphate dehydrogenase (*GAPDH*) were as follows: *Nrf2*, 26 cycles of 94°C for 1 min; 60°C for 1 min; 72°C for 1 min, *HO-1*, 26 cycles of 94°C for 1 min; 60°C for 1 min; 72°C for 1 min, *NQO1*, 26 cycles of 94°C for 1 min; 58°C for 1 min, *GAPDH*, 26 cycles of 94°C for 1 min; 56°C for 2 min; 72°C for 2 min. The primer pairs (Bionics, Seoul, South Korea) were as follows: *Nrf2*, 5′-CGGTATGCAACAGGACATTG-3′ and 5′-ACTGGTTGGGGTCTTCTGTG-3′; *HO-1*, 5′-CAGGCAGAGAATGCTGAGTTC-3′ and 5′-GATGTTGAGCAGGAACGCAGT-3′; *NQO1*, 5′-CGCAGACCTTGTGATATTCCAG-3′ and 5′-CGTTTCTTCCATCCTTCCAGG-3′; *GAPDH*, 5′-TGAAGGTCGGTGTCAACGGATTTGGC-3′ and 5′-CATGTAGGCCATGAGGTCCACCAC-3′ (forward and reverse, respectively). Amplification products were resolved by 1.0% agarose gel electrophoresis, stained with ethidium bromide, and photographed under ultraviolet light.

### Site-directed mutagenesis

Site-directed mutagenesis was performed by PCR using DNA primers obtained from Bioneer (Daejeon, Korea) with single-, double- or triple-base mismatches, resulting in the desired amino acid substitution according to the manufacturer's instruction (Intron Biotechnology, Sungnam, Korea, Cat. No. 15071). The correct sequence of all constructs was confirmed by sequencing (Cosmo Genetech, Seoul, Korea).

### Generation of stable cells expressing Keap1 mutant constructs

pBabe parental vector (pBabe-puro-HA-VHL) was obtained from Addgene (Cambridge, MA, USA). PCR-amplified wild-type Keap1, Keap1-C151S, -C273S, or –C288S cDNA was subcloned into the parental vectors. Retroviral-mediated stable expression of each vector was generated in HEK293T cells. Recombinant retroviruses were produced as described previously [26], [27], [28]. Briefly, HEK293T cells were transiently transfected with 8 µg of each retroviral construct and 4 µg of helper vectors (gagpol and VSVG). At 24 h after transfection, the media were changed to RPMI 1640 containing 10% FBS, and at 48 h, the virus containing media were collected, filtered (0.45 µm), and supplemented with 8 µg/ml polybrene (Millipore, Billerica, MA, USA). Target cells were incubated for 24 h with the retrovirus-containing solution. Selection with puromycin (2 µg/ml) was initiated at 24 h and continued for 1 week.

### Enzymatic in-gel digestion

Human recombinant Keap1 protein was incubated at 37°C for 0.5 h with DATS (100 µM) or vehicle and was separated by NuPAGE® 4–12% *bis*-Tris Gel (Invitrogen, Carlsbad, CA, USA). After separation, the gel was stained with GelCode® Blue Stain Reagent (Thermo scientific, Rockford, IL, USA), and the gel pieces containing proteins were destained with 50% acetonitrile (ACN) containing 50 mM NH_4_HCO_3_ and vortexed until Coomassie brilliant blue (CBB) was completely removed. These gel pieces were then dehydrated in 100% ACN and vacuum-dried for 20 min with SpeedVac®. For the digestion, gel pieces were alkylated using 55 mM iodoacetamide in 50 mM NH_4_HCO_3_ for 0.5 h in dark. Finally, each gel piece was treated with 10 ng/µl sequencing grade chymotrypsin (Roche, Mannheim, Germany) in digestion buffer (100 mM Tris-HCl, 10 mM CaCl_2_, pH 7.8) at 25°C for overnight. Following digestion, peptide fragments were extracted with 5% formic acid in 50% ACN solution at room temperature for 20 min. Supernatants were collected and dried with SpeedVac®. Samples resuspended in 0.1% formic acid were purified and concentrated using C18 ZipTips (Millipore, Billerica, MA, USA) before mass spectroscopic (MS) analysis.

### Analysis by nano-LC-ESI-MS/MS

The products of in-gel digestion of recombinant Keap1 pre-incubated with or without DATS were loaded onto a fused silica microcapillary column (12-cm×75 µm) packed with C18 reverse-phase resin (5 µm, 200 Å). LC separation was conducted under a linear gradient: a 3–40% solvent B (ACN containing 0.1% formic acid) gradient (solvent A; distilled water containing 0.1% formic acid), with a flow rate of 250 nl/min, for 1 h. The column was directly connected to LTQ linear ion-trap mass spectrometer (Thermo scientific, Rockford, IL, USA) equipped with a nano-electrospray ion source. The electrospray voltage was set at 1.95 kV, and the threshold for switching from MS to MS/MS was 500. The normalized collision energy for MS/MS was 35% of main radio frequency amplitude (RF) and the duration of activation was 30 ms. All spectra were acquired in a data-dependent scan mode. Each full MS scan was followed by five MS/MS scan (?) scans ranging from the most intense to the fifth intense peaks of full MS scan. Repeat count of peak for dynamic exclusion was 1, and its repeat duration was 30 s. The dynamic exclusion duration was set for 180 s and width of exclusion mass was ±1.5 Da. The list size of dynamic exclusion was 50. The acquired LC-ESI-MS/MS fragment spectra were searched in the BioWorksBrowser™ (version Rev. 3.3.1 SP1, Waltham, MA, USA) with the SEQUEST search engines against the data in FASTA format generated from Keap1, transcript variant 1, mRNA (NCBI accession number NM_203500) in National Center for Biotechnology Information (http://www.ncbi.nlm.nih.gov/). The conditions for the search were as follows; chymotrypsin as enzyme specificity, a permissible level for two missed cleavages, peptide tolerance; ±2 amu, a mass error of ±1 amu on fragment ions and variable modifications of carbamidomethylation of cysteine (+57 Da), oxidation of methionine (+16 Da) and DATS-induced oxidative modification of cysteine (+72.1 Da) residues.

### Statistical analysis

When necessary, data were expressed as means ± SD of at least three independent experiments, and statistical analysis for single comparison was performed using the Student's *t*-test. The criterion for statistical significance was *, ** and *** as *p*<0.05, 0.01 and 0.001, respectively.

## Results

### DATS induces the expression of HO-1 and NQO1 in cultured AGS cells as well as in mouse stomach *in vivo*

**Figure 1:**
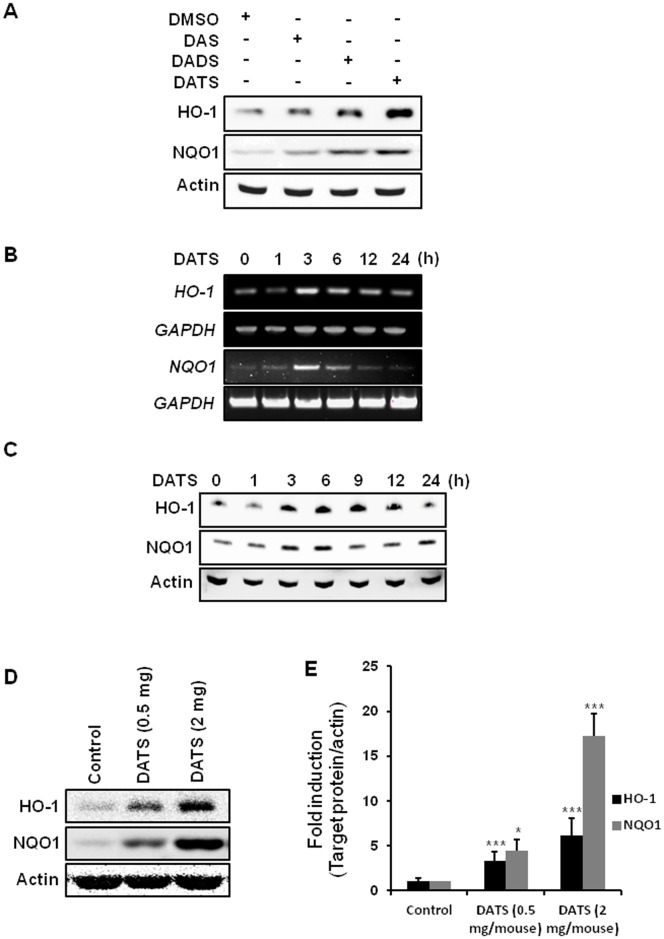
DATS induces the expression of HO-1 and NQO1. (A) AGS cells were incubated with DAS, DADS, or DATS (20 µM each) for 6 h, and the protein expression of HO-1 and NQO1 was determined by Western blot analysis. Actin was detected to ensure equal protein loading. (B) Total RNA was isolated from cells treated with or without DATS for indicated duration and was analyzed by RT-PCR for detecting the levels of *HO-1* and *NQO1* mRNA, as described in Metarials and Methods. *GAPDH* was used as a control for equal lane loading. (C) The expression of HO-1 and NQO1 in AGS cells incubated in the presence or the absence of DATS (20 µM) for the indicated duration was assessed by Western blot analysis. (D) C57BL/6 mice were given DATS (0.5 or 2 mg per mouse) suspended in sodium-CMC or vehicle alone as described in Metarials and Methods. After 2 weeks, stomach tissues were collected, and the protein lysate was prepared. The effect of DATS on the expression of HO-1 and NQO1 was assessed by Western blot analysis. (E) Data are represented as fold induction of protein expression. Data are means ± SEM (n = 5); **, *** Significantly different as compared to control (*p*<0.01, 0.001 respectively).

We initially attempted to compare the effects of DAS, DADS and DATS on the expression of HO-1 and NQO1, two representative antioxidant enzymes, in AGS cells. Incubation of cells with each of these OSCs at a concentration of 20 µM for 6 h revealed that DATS was most potent in inducing expression of HO-1 and NQO1 at both transcriptional and translational levels (Fig. 1A). Treatment of AGS cells with DATS (20 µM) induced the mRNA (Fig. 1B) and protein (Fig. 1C) expression of HO-1 and NQO1 in a time-dependent manner. The protein expression of HO-1 and NQO1 following DATS treatment peaked at 6 h. In another experiment, administration of DATS (0.5 or 2 mg/mouse) by gavage every other day for two weeks also significantly induced the expression of HO-1 and NQO1 in the mouse stomach in a dose-dependent manner (Fig. 1D and 1E).

### DATS stimulates the nuclear accumulation of Nrf2 in AGS cells and the mouse stomach

**Figure 2:**
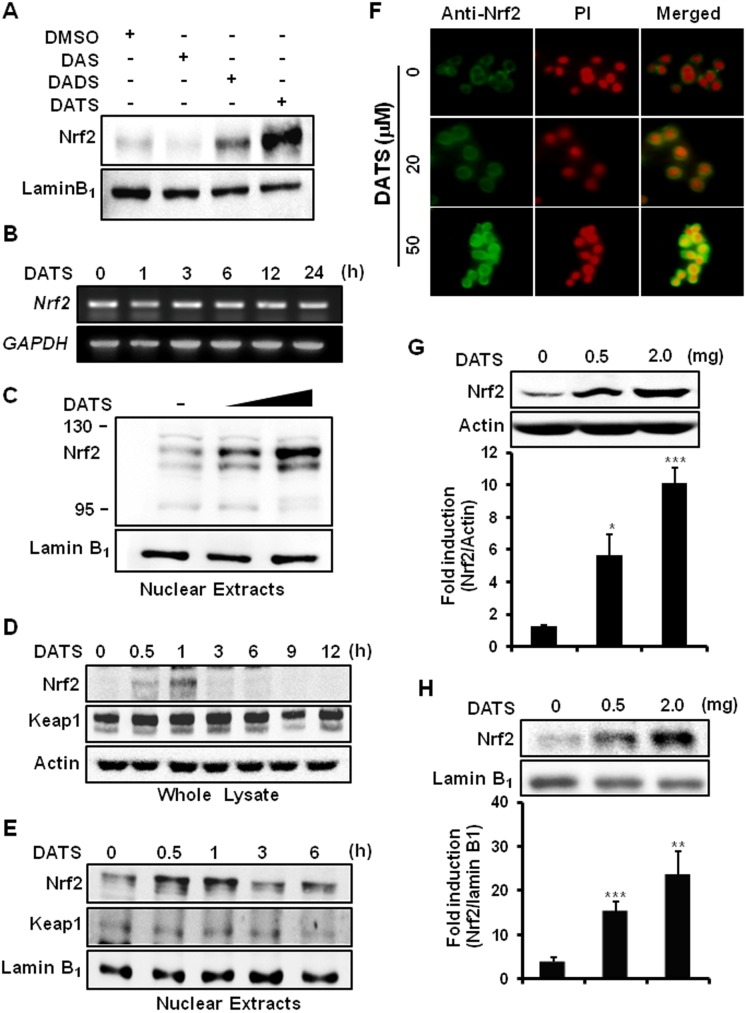
DATS induces the nuclear accumulation of Nrf2 *in vitro* and *in vivo*. (A) AGS cells were treated with DAS, DADS, and DATS (20 µM of each). After 1 h, the cells were harvested, and fractionated to prepare the cytosolic and nuclear extracts. The nuclear level of Nrf2 was determined by Western blot analysis. Lamin B_1_ was used to ensure the purity and equal loading of nuclear protein. (B) AGS cells treated with or without DATS (20 µM) were harvested at the indicated intervals, and total RNA was prepared. The RNA samples were analyzed by RT-PCR for detecting the levels of *Nrf2* mRNA, as described in Metarials and Methods. *GAPDH* was used as a control for equal loading. (C) Nuclear extracts prepared from cells treated with DATS (5 or 20 µM) were subjected to Western blot analysis for detecting the nuclear localization of Nrf2. (D) Cells, treated with DATS (20 µM) for the indicated time periods, were lysed and the protein expression of Nrf2 and Keap1 in the whole cell lysate was determined by Western blot analysis. (E) Upon incubation with DATS (20 µM) for the indicated time, nuclear extract was prepared and immunoblotted to determine the nuclear localization of Nrf2 and Keap1. (F) AGS cells were treated with DATS (5 or 20 µM) for 1 h. The immunofluorescence staining of Nrf2 was conducted as described in Metarials and Methods. (G–H) Data are represented as fold induction of Nrf2 expression against control. Error bars represent SEM. *Columns*, means (n = 3); *, **, and *** Significantly different from that of control (*p*<0.05, 0.01, and 0.001, respectively) (G) The expression of Nrf2 from whole lysate of mouse gastric tissue prepared as described for Fig. 1D was assessed by Western blot analysis. (H) The gastric tissues of mice treated with DATS or vehicle were fractionated, and nuclear localization of Nrf2 was determined by Western blot analysis.

Since Nrf2 plays an important role in the transcriptional regulation of HO-1 and NQO1 expression, we first examined the relative potency of DAS, DADS and DATS in terms of inducing the nuclear localization of Nrf2 in AGS cells. Consistent with the relative effects on HO-1 and NQO1 expression, DATS most strongly induced the nuclear translocation of Nrf2 (Fig. 2A). However, DATS failed to alter the Nrf2 mRNA expression (Fig. 2B). The DATS-induced nuclear accumulation of Nrf2 was concentration-dependent (Fig. 2C). DATS treatment resulted in a transient increase in the total (Fig. 2D) and the nuclear (Fig. 2E) levels of Nrf2. The nuclear accumulation of Nrf2 was further confirmed by immunocytochemistry (Fig. 2F). We also examined the effect of orally administered DATS on Nrf2 activation in the mouse stomach *in vivo*. Administration of DATS (0.5 or 2 mg/mouse) every other day for two weeks by gavage elevated the levels of Nrf2 in both the whole lysate (Fig. 2G) and nuclear extract (Fig. 2H) prepared from the stomach tissues of treated mice.

### The DATS-induced expression of HO-1 and NQO1 is dependent on the activation of Nrf2

**Figure 3:**
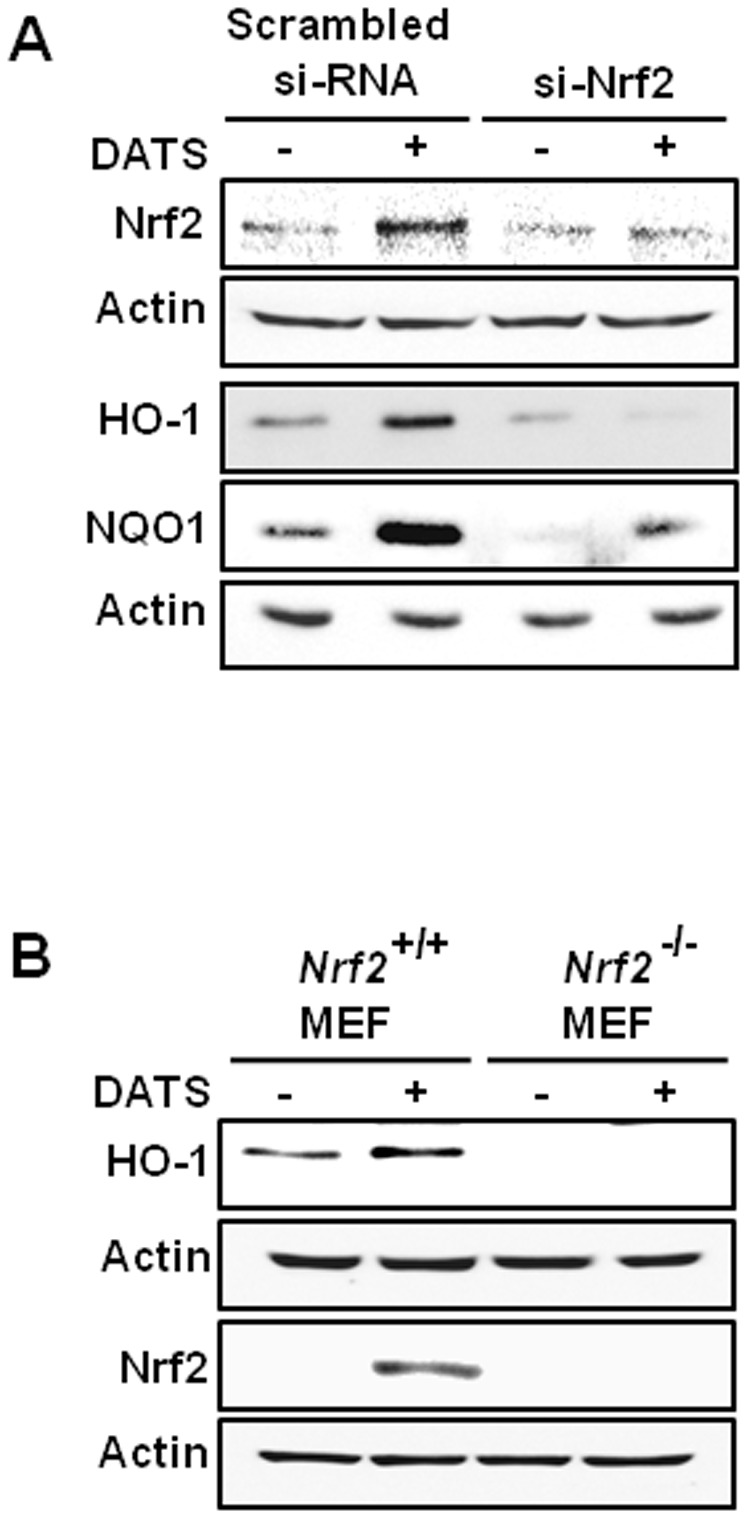
Knockdown of Nrf2 abrogates DATS-induced expression of HO-1 and NQO1. Cells transfected with *Nrf2*-siRNA or *nrf2*-null MEFs were incubated with 20 µM of DATS for 1 h or 6 h, respectively, to determine the levels of Nrf2 or its target protein HO-1 and NQO1. Cells were harvested and the protein lysate was subjected to Western blot analysis. The incubation conditions and other experimental details are described in Experimental procedures.

We then examined whether Nrf2 does mediate the DATS-induced expression of HO-1 and NQO1. AGS cells were transiently transfected with control siRNA or Nrf2 siRNA (si-Nrf2) and incubated with DATS or vehicle alone. As compared to cells transfected with scrambled siRNA, DATS-induced expression of Nrf2, HO-1 and NQO1 was abrogated in cells transfected with si-Nrf2 (Fig. 3A). To verify the role of Nrf2 in DATS-induced HO-1 expression, we also utilized embryonic fibroblasts obtained from the Nrf2 wild-type and knockout mice. Treatment with DATS failed to induce the expression of HO-1 as well as Nrf2 in fibroblasts from *Nrf2^−/−^* mice (Fig. 3B).

### ROS are involved in DATS-induced activation of Nrf2 and expression of HO-1 and NQO1 in AGS cells

**Figure 4:**
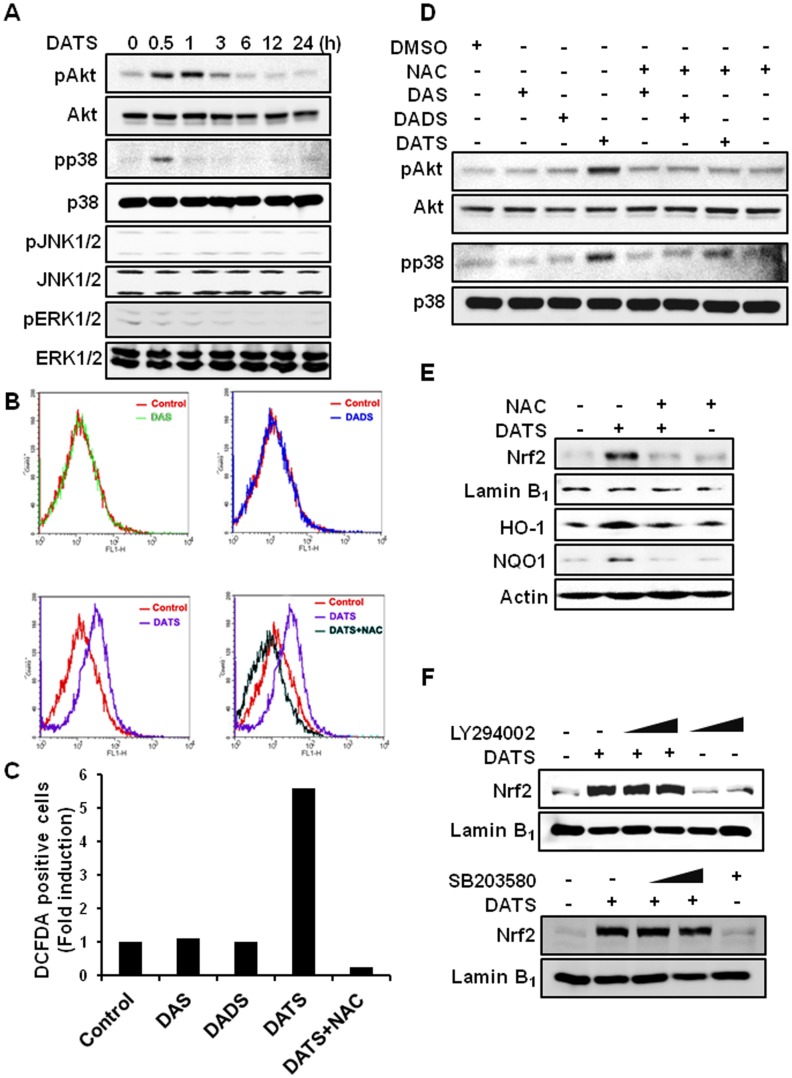
ROS, but not Akt or p38 MAPK, are involved in DATS-induced Nrf2 activation. (A) AGS cells were incubated with DATS (20 µM) for the indicated time, and the expression of both phosphorylated and total forms of Akt, p38 MAPK, JNK1/2, and ERK1/2 were measured by Western blot analysis. (B) AGS cells were incubated with 20 µM of DAS, DADS, and DATS in the presence of NAC (1 mM) for 3 h. ROS accumulation was determined by the DCF-DA assay as described in Metarials and Methods. (C) Data are represented as fold induction of the number of DCF-DA positive cells (n = 5) calculated from Fig. 4B. (D) AGS cells treated with DAS, DADS, or DATS (20 µM, each) for 0.5 h with or without NAC (1 mM) were analyzed by Western blotting to detect phosphorylated and total Akt and p38 MAPK. (E) Cells treated with DATS (20 µM) in the absence or the presence of NAC (1 mM) were harvested to determine the nuclear level of Nrf2 and the protein expression of HO-1 and NQO1. (F) AGS cells were pre-treated with 12.5 and 25 µM of LY 294002, an inhibitor of PI3K/Akt, or SB203580, a pharmacological inhibitor of p38 MAPK, followed by DATS (20 µM) treatment for 1 h. The nuclear protein extract was subjected to immunoblot analysis for the detection of Nrf2 expression.

It has been reported that activation of MAPKs or Akt is involved in Nrf2-mediated gene expression [15], [16], [17], [18]. We examined whether DATS could induce the activation of these upstream kinases. Treatment with DATS for 30 min increased the phosphorylation of Akt and p38 MAPK, but not ERK and JNK, in AGS cells (Fig. 4A). Since ROS are known to induce phosphorylation of Akt and p38 MAPK, we examined the effects of DAS, DADS and DATS on the generation of ROS. While treatment of AGS cells with DAS or DADS did not induce ROS, incubation with DATS significantly increased the level of ROS as assessed revealed by the enhanced DCF-derived fluorescence, which was abrogated by pretreatment with NAC (Fig. 4B and 4C). Likewise, only DATS induced the phosphorylation of Akt and p38 MAPK. NAC attenuated DATS-induced phosphorylation of Akt and p38 MAPK (Fig. 4D). Moreover, incubation of cells with DATS in the presence of NAC diminished DATS-induced nuclear localization of Nrf2 as well as the expression of HO-1 and NQO1 (Fig. 4E). To verify the role of Akt and p38 MAPK in DATS-induced activation of Nrf2, we treated cells with DATS in the presence of an inhibitor of Akt (LY2940002) or p38 MAPK (SB203580). However, pharmacological inhibition of both kinases failed to affect DATS-induced Nrf2 activation in AGS cells (Fig. 4F).

Although DATS apparently induce intracellular ROS accumulation it is still unclear how it generates the ROS and which type of ROS is produced. As DCF is a non-specific ROS detecting fluorescent dye [29], further investigation using probes that can detect specific reactive species will be necessary.

### The activation of Nrf2 and the induction of HO-1 and NQO1 expression by DATS are likely to be mediated through modification of the Keap1 Cys288 residue

**Figure 5:**
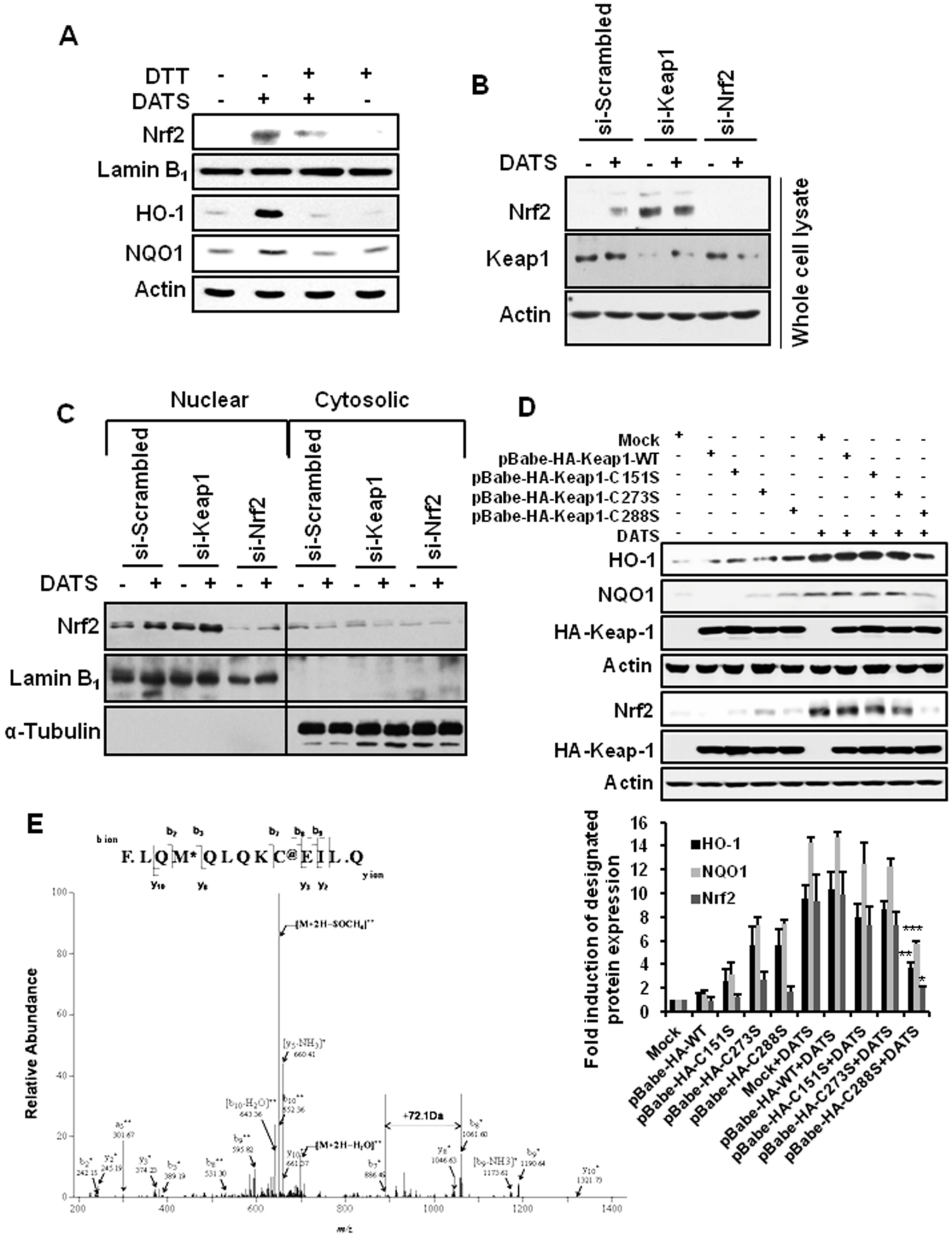
The thiol modification of Keap1 Cys288 is responsible for DATS-induced activation of Nrf2 signaling. (A) Cells were incubated with DATS (20 µM) in the presence or the absence of DTT (50 µM). Both the nuclear localization of Nrf2 and the expression of HO-1 and NQO1 were determined by Western blot analysis. (B) AGS cells were transfected with scrambled (si-scrambled), Keap1 siRNA (si-Keap1) or Nrf2 siRNA (si-Nrf2) for 24 h and incubated with DATS (20 µM) for additional 2 h. Total Nrf2 expression was detected by immunoblot analysis. (C) Cells were treated with DATS (20 µM) after transfection with siRNAs targeting Keap1 or Nrf2. The nuclear and cytosolic level of Nrf2 was detected. (D) AGS cells stably expressing Mock vector, HA-WT-Keap1, HA-Keap1-C151S, HA-Keap1-C273S or HA-Keap1-C288S were generated as described in Experimental procedures. Each of mutant cell lines was incubated with DATS (20 µM) for 1 h or 6 h to determine the expression of Nrf2 or HO-1 and NQO1, respectively. HA-Keap1 was used to ensure the equal expression of the mutant vectors. Data are shown as fold induction of protein expression against Mock. Error bars represent SEM. *Columns*, means (n = 10); *, **, and *** Significantly different from that of mock (*p*<0.05, 0.01, and 0.001 respectively) (E) Human recombinant Keap1 protein was incubated with DATS (100 µM) for 0.5 h and subjected to in-gel digestion. Digested peptide fragments were subjected to mass spectrometry as described in Experimental procedures.

Under physiologic conditions, Nrf2 is present in the cytoplasm as an inactive complex with Keap1. Reactive cysteine residues present in Keap1, particularly Cys151, Cys273 and Cys288, function as sensors for electrophilic and oxidative stresses [14], [19], [20], [21]. To elucidate the mechanisms underlying DATS-induced Nrf2 activation, we examined the possible effect of DATS on thiol modification of critical cysteine residues present in Keap1. The effects of DATS on the nuclear translocation of Nrf2 and the expression of HO-1 and NQO1 were abrogated when cells were pre-treated with a thiol reducing agent, DTT (Fig. 5A). To confirm that Keap1 is the target of DATS, we transfected cells with scrambled or Keap1 siRNA prior to treatment with DATS. Compared to scrambled siRNA, transfection with Keap1 siRNA caused a marked increase in the total Nrf2 expression, which remained almost unchanged upon treatment with DATS (Fig. 5B). Transfection with Nrf2 siRNA was conducted to ensure Keap1 siRNA transfection efficiency. Under the same experimental conditions, we examined the nuclear and cytosolic levels of Nrf2. Compared to scrambled siRNA, transfection with Keap1 siRNA enhanced nuclear localization of Nrf2, which was further increased upon treatment with DATS (Fig. 5C). To identify the critical Keap1 cysteine residue as a target of DATS, we constructed HA-tagged retroviral vectors harboring site-directed mutation of Cys151, 273 or 288 to serine (C151S, C273S and C288S, respectively). Stable expression of exogenous human wild type Keap1 and its mutants was validated by immunoblotting of HA. DATS failed to activate Nrf2 as well as induce expression of HO-1 and NQO1 in cells transfected with the Keap1-C288S construct, whereas both events were not much affected in the other two mutant (C151S and C273S) cell lines by the same treatment (Fig. 5D). This finding suggests that DATS may directly interact with Cys288 of Keap1. We, therefore, performed a mass spectrometric analysis to determine whether DATS could directly modify the Cys288 residue of Keap1. Human recombinant Keap1 incubated with DATS was digested with chymotrypsin, and analyzed by tandem mass spectrometry. An increment of 72.1 Da, the molecular mass of mono-allyl mono-sulfide (a cleaved form of DATS), in the peptide fragment containing Cys288 was observed (Fig. 5E).

## Discussion

Gastric cancer is one of the most common types of malignancies and also the second leading cause of cancer-related deaths in the world [30], [31]. Oxidative stress is implicated in many human ailments including gastric cancer. ROS generated from various sources damage cellular macromolecules, such as proteins, lipids and nucleic acids, thereby contributing to the neoplastic transformation [32], [33], [34], [35]. However, living organisms exploit several modes of self-defensive mechanisms in order to protect against the deleterious effects of excessive ROS production or accumulation. Some hormetic chemopreventive phytochemicals with antioxidant properties mimic this innate strategy of body's self-protection against oxidative stress [36]. Garlic-derived OSCs have been known to possess cancer chemopreventive potential, which is partly ascribed to their antioxidant properties. However, the detailed molecular mechanisms by which the OSCs exert antioxidant effects remain still unanswered.

Previously, Fisher *et al.* reported that DAS was more potent than other allyl sulfides in inducing the NQO1 mRNA expression and the ARE activity when administered to WKY rats [37]. In our study, the relative capability of different OSCs to activate Nrf2 and to induce the expression of HO-1 and NQO1 in AGS cells was in the order of DATS>DADS>DAS. However, the possibility of masking or stimulating effects of impurities present in these OSCs on their relative efficacy cannot be excluded. The different potency of OSCs in the induction of antioxidant enzymes appears to be associated with the relative stability/reactivity of their carbon-sulfur bond [38]. It has been reported that OSCs differentially react with reduced glutathione (GSH): the more sulfur atoms contained in an OSC, the higher antioxidant activity the compound exerts [39].

The transcriptional activation of genes encoding antioxidant or cytoprotective proteins is largely regulated by the redox-sensitive transcription factor Nrf2, which in the balanced redox state of the cells remains inactive by forming a complex with an inhibitory protein Keap1 in the cytoplasm. Upon electrophilic/oxidative stress, Nrf2 is dissociated from Keap1 and translocates to the nucleus. The reduced ability of DATS to induce the expression of HO-1 and NQO1 in cells lacking Nrf2 indicates that DATS-mediated expression of these antioxidant enzymes is Nrf2-dependent.

There have been harsh debates on the exact mechanism of Nrf2 inducers. Nrf2, a stress responsive transcription factor, is constantly degraded through polyubiquitination by the Keap1-Cul3 E3 ubiquitin ligase complex. When cells are challenged with stresses, Nrf2 evades polyubiquitination by the Keap1-Cul3 E3 ubiquitin ligase complex and accumulates in the nucleus to induce the transcription of stress-response genes. Two plausible mechanisms have been proposed to explain the Nrf2 activation by various chemopreventive agents: one is the phosphorylation of Nrf2 by kinases, such as MAPKs or Akt, and the other is Keap1 inactivation through modification of critical cysteines of the Keap1 protein. Numerous studies have been implemented to elucidate the association between Nrf2 and MAPKs. However, Sun *et al.* pointed out that MAPKs might make a limited contribution to the activation of Nrf2 and that Nrf2 stability is not likely to be altered by its phosphorylation [40].

To explore the mechanisms underlying the Nrf2 activation by DATS, we initially focused on several upstream kinases previously reported to be involved in the activation of Nrf2 signaling. Our finding that DATS, but not DAS or DADS, generates ROS suggests that the DATS-induced ROS production and the subsequent activation of upstream kinases may lead to the elevated transcriptional activity of Nrf2. In line with this notion, pre-incubation of cells with NAC attenuated not only DATS-induced generation of ROS and the phosphorylation of Akt and p38 MAPK, but also the nuclear localization of Nrf2 and the expression of HO-1 and NQO1. However, our study revealed that the blockade of Akt or p38 MAPK had no effect on DATS-induced Nrf2 nuclear localization. This result implies that phosphorylation of Nrf2 by ROS generated by DATS, if any, is unlikely to be a principal mechanism responsible for DATS-induced Nrf2 activation. However, the possibility that DATS-induced ROS can activate Nrf2 through oxidation of critical cysteine residue, especially Cys288 that function as a redox sensor, cannot be ruled out (*vide infra*).

Kobayashi *et al.*, have reported that the activity of Nrf2 is mainly regulated by the Keap1-E3 ubiquitin ligase complex [41]. ROS or electrophiles can structurally alter reactive cysteines in Keap1, thereby attenuating the function of Keap1 in sequestering Nrf2 in the cytoplasm. As an initial approach to test the possibility of DATS-mediated Nrf2 activation through direct Keap1 cysteine thiol modification, we utilized NAC and DTT capable of interfering with cysteine modification by oxidants and electrophiles. It has been reported that DATS can react with molecules containing reduced thiols, such as GSH [42]. Since NAC acts not only as a GSH precursor, but also as a nucleophile, it may form a conjugate with DATS, thereby limiting the DATS access to Keap1 and consequently minimizing Keap1 cysteine thiol modification. DTT, a prototypic thiol reducing agent, also attenuated the DATS-induced Nrf2 activation and expression of HO-1 and NQO1. DTT is a well-known reducing agent due to the presence of two thiol groups, which therefore can reduce a disulfide bond through two sequential thiol-disulfide exchange reactions. It is unclear whether NAC and DTT work in an exactly same way or not in our system but we hypothesized that DTT could block the activity of DATS at least by keeping Keap1 thiol in a reduced state, thereby hampering interaction between DATS and critical Keap1 cysteine.

There are three critical thiol residues (Cys151, Cys273, Cys288) present in Keap1 that have been speculated to regulate the level of Nrf2. In our current study, the mutation of Cys288 in Keap1 abolished the DATS-induced expression of HO-1, and NQO1 as well as Nrf2 activation, corroborating that DATS may interact with this cysteine directly. The mass spectral analysis of human wild-type Keap1 recombinant protein incubated with DATS revealed a mass increment of 72.1 Da corresponding precisely to the molecular weight of mono-allyl mono-sulfide in the peptide fragment of Keap1 containing Cys288, which is indicative of direct interaction of DATS with this particular cysteine. Although Cys151 of Keap1 has largely been reported to function as an essential electrophilic sensor mediating the antioxidant activity of a myriad of Nrf2 inducers [14], [43], [44], little is known about the role of the Keap1 Cys288 residue in Nrf2 activation by electrophiles. Intriguingly, our observation that DATS-induced activation of Nrf2, and concurrent expression of HO-1 and NQO1 are abated in cells harboring Keap1 C288S mutant, suggests that the Cys288 residue of Keap1 can also serve as a critical sensor of electrophilic assaults.

Since Cys288 exists in an intervening region (IVR) of Keap1, it has been suggested that Cys288 along with Cys273 mediates an interaction between IVR region of Keap1 and Neh2 domain, especially DLG and ETGE motif, of Nrf2 and is hence responsible for the repression of Nrf2 [41], [45]. Ogura *et al.* have reported that oxidative and electrophilic stress can cause modification of Cys273 and Cys288 residues, thereby inducing conformational changes in IVR and the neighboring DC domain (double glycine repeat and C-terminal region), and eventually alter the substrate-binding ability of Keap1 [46]. By contrast, it has been suggested that the modification of Cys151 may not influence the recognition of Nrf2, but may rather impair the E3 ligase complex, based on the crystal structure of Keap1 [46]. Different cysteine residues of Keap1, including Cys273 and Cys288, are considered to be sensed differentially by distinct electrophiles. We speculate that mono-allyl mono-sulfide, a cleaved product of chemically reactive DATS, may interact preferentially with Keap1 Cys288, thereby inducing Nrf2 activation. Further studies will be necessary to clarify Cys288 as a *bona fide* target of DATS *in vivo*.

In conclusion, DATS induces the transcriptional activation of Nrf2 and subsequent expression of HO-1 and NQO1 by directly modifying Keap1, presumably at its Cys288 residue that acts as a sensor of oxidants and electrophiles. DATS-derived mono-allyl mono-sulfide is considered to react with this particular Keap1 cysteine to form *S*-allyl mercaptocysteine, which may explain how DATS, among OSCs, exhibits remarkable capability in inducing defensive gene expression through Nrf2 activation.
